# Living grass mulching improves soil enzyme activities through enhanced available nutrients in citrus orchards in subtropical China

**DOI:** 10.3389/fpls.2022.1053009

**Published:** 2022-12-08

**Authors:** Na Wang, Le Li, Mengmeng Gou, Zunji Jian, Jianwen Hu, Huiling Chen, Wenfa Xiao, Changfu Liu

**Affiliations:** ^1^ Key Laboratory of Forest Ecology and Environment, Nation Forestry and Grassland Administration, Ecology and Nature Conservation Institute, Chinese Academy of Forestry, Beijing, China; ^2^ Research Institute of Tropical Forestry, Chinese Academy of Forestry, Guangzhou, China; ^3^ Co–Innovation Center for Sustainable Forestry in Southern China, Nanjing Forestry University, Nanjing, China

**Keywords:** living grass mulching, soil enzyme activity, soil nutrient, mulching age, soil layer, orchard

## Abstract

Living grass mulching (LGM) is an important orchard floor management that has been applied worldwide. Although LGM can effectively enhance soil nutrient availability and fertility, its effects on microbial-mediated soil nutrient cycling and main drivers are unclear. Meanwhile, the variation of enzyme activities and soil nutrient availability with LGM duration have been rarely studied. This study aims to explore the effects of mulching age and soil layer on enzyme activities and soil nutrients in citrus orchards. In this study, three LGM (*Vicia villosa*) treatments were applied, i.e., mulching for eight years, mulching for four years, and no mulching (clean tillage). Their effects on the enzyme activities and soil nutrients were analyzed in different soil layers of citrus orchards in subtropical China, i.e., 0-10, 10-20, and 20-40 cm. Compared to clean tillage, mulching for four years had fewer effects on enzyme activities and soil nutrients. In contrast, mulching for eight years significantly increased available nitrogen (N), phosphorus (P) nutrients, *β*-glucosidase, and cellobiohydrolase activities in the soil layer of 0-20 cm. In the soil layer of 0-40 cm, microbial biomass carbon (C), N, P, N-acetylglucosaminidase, leucine aminopeptidase, and acid phosphatase activities also increased (*P* < 0.05). Mulching for eight years significantly promoted C, N, and P-cycling enzyme activities and total enzyme activities by 2.45-6.07, 9.29-54.42, 4.42-7.11, and 5.32-14.91 times, respectively. Redundancy analysis shows that mulching treatments for eight and four years had soil layer-dependent positive effects on soil enzyme activities. Microbial C and P showed the most significant positive correlation with enzyme activities, followed by moisture content, organic C, and available N (*P* < 0.05). Available nutrients contributed almost 70% to affect enzyme activities significantly and were the main drivers of the enzyme activity variation. In summary, LGM could improve soil enzyme activities by increasing available nutrients. The promotion effect was more significant under mulching for eight years. Therefore, extending mulching age and improving nutrient availability are effective development strategies for sustainable soil management in orchard systems. Our study can provide valuable guidelines for the design and implementation of more sustainable management practices in citrus orchards.

## Introduction

Orchards have been widely maintained worldwide and have become an essential part of agriculture owing to the tremendous economic value of the fruit (Rey, 2011; Zhao et al., 2021). The acreage of orchards has increased by approximately 22% since 2000 worldwide (FAO, 2020). Soil is the foundation of ensuring orchard productivity and promoting ecosystem stability. Agricultural management regimes largely affect soil properties and biochemical processes (Jia et al., 2022). Orchard management practices vary across regions, resulting in differential impacts on soil quality (e.g., physicochemical and biochemical properties) (Demestihas et al., 2017; Xiang et al., 2023). China has the largest orchard area in the world (Xiang et al., 2022). Although orchards have contributed significantly to improving the total vegetation coverage in China, understory management of orchards is still lagging (Wei et al., 2017). Clean tillage (total weeding control) is a popular orchard floor management practice in China (Wang et al., 2015). Implementing clean tillage management in orchards can speed up soil organic matter’s mineralization and decomposition and exacerbate the decrease of enzyme activities and soil microbial community diversity (Wang et al., 2009; Vignozzi et al., 2019; Xiang et al., 2022). Optimal management practices, such as living grass mulching (LGM), have been promoted to curb soil degradation in orchards (Rumpel et al., 2020). However, farmers and decision-makers have not realized its potential impacts on the orchard ecosystems, resulting in the slow implementation of optimal practices (Wei et al., 2017). Therefore, a critical assessment of soil characteristics responding to orchard floor management is necessary to realize sustainable utilization of orchard soils.

LGM is a soil management method that replaces whole-garden or inter-row bare soil with sod cultivation (Atucha et al., 2013; Wang et al., 2015; Taguas et al., 2017). Previous research has shown that LGM may alter many aspects of soil properties in orchards, such as soil physical properties (Haruna et al., 2020), soil organic carbon (SOC) stocks (Xiang et al., 2022), soil nutrient contents (Wei et al., 2017), soil biological activities (Ramos et al., 2011), and soil microbial community composition (Wang et al., 2022). Thus, LGM is very likely to affect the ecosystem functions of orchards (Wei et al., 2018). Soil enzymes can directly mediate the catabolism of soil organic and mineral components and are crucial in biogeochemical cycles within terrestrial ecosystems (Margida et al., 2020; Liu et al., 2022). Soil enzyme activity is more sensitive to soil quality changes compared with physicochemical properties (e.g., soil nutrient content and organic matter) (Luo et al., 2018). It can be considered an early warning indicator of soil system changes (Utobo and Tewari, 2015). LGM has been reported to increase the activities of soil enzymes, such as urease and phosphatase activity (Xiang et al., 2023). However, some studies have reported opposite results or no effects (Solanki et al., 2019; Adetunji et al., 2021). Kumar et al. (2022) also pointed out that the positive effects of mulching measures on soil enzyme activity could be enzyme-specific. Furthermore, LGM affects soil enzyme activity by changing soil properties (e.g., temperature, pH, soil bulk density, water content, and nutrient content) (Burns et al., 2016). LGM can improve the exogenous input of soil organic matter and enzyme activity, thus accelerating organic matter degradation and soil nutrient mineralization and improving soil nutrient levels. This indicates that enzyme activity has a positive correlation with organic matter (Zheng et al., 2018). However, exceptions exist (Sun et al., 2021). Burns et al. (2013) found that increased soil nutrients promoted nutrient uptake by microorganisms and thus reduced related catalytic enzyme activities. This indicates that soil enzyme activity is mainly affected by soil nutrient availability. Moreover, a relatively high element content in soils can also promote other elements’ use by extracellular enzymes (Sinsabaugh, 2010). Therefore, inter-element coupling increases the difficulty in determining the mechanism of enzyme activity changes under orchard floor management. These inconsistent results highlight the call for more cases to better understand the soil biological properties in orchards in response to LGM.

The effects of LGM on soil biological properties may be a long-term process. Due to strong anthropogenic disturbance, short-term LGM may not cause significant changes in orchard soil properties (Wang et al., 2020a). Nevertheless, most studies have focused on the effects of short-term LGM on orchard soil biological properties. The use of LGM for a different number of years in the orchard has been rarely studied. Moreover, various biotic and abiotic factors affecting soil enzyme activity vary with soil layers (Sun et al., 2021). Generally, enzyme activity decreases with increasing soil layer depth (Stone et al., 2014). However, orchard disturbances (e.g., fertilization, irrigation, and understory maintenance) have more direct and significant effects on the topsoil than on the subsoil (Sun et al., 2021). Some studies have reported that enzyme-associated mineralization rates of deep soil mineral nitrogen (N) or carbon (C) in subsoil were close to or higher than those in topsoil under environmental variations (Schnecker et al., 2015; Wang et al., 2019). Hence, enzyme activity variations in different soil layers of orchards due to LGM remain unclear. Evaluating the dynamics of enzyme activities and soil nutrients under different mulching ages and soil layers is necessary in order to better understand the biochemical processes under LGM.

Citrus is the fruit tree with the largest planting area in the world. It can promote regional economic development and ecological environment (Tu et al., 2021). The Three Gorges Reservoir area (TGRA) in China is one of the optimal citrus production regions worldwide due to its unique natural resources and ecological conditions (Xia et al., 2015). Currently, clean tillage is commonly adopted for citrus orchard floor management, resulting in soil degradation problems such as soil compaction and decreasing organic matter (Liang and Li, 2019). This study aims to provide a beneficial biological approach for improving soil nutrient cycling efficiency and quality in citrus orchards. In this study, *Vicia villosa* (a leguminous plant) was selected as mulching grass to investigate enzyme activities and soil nutrients in different soil layers (0-40 cm) under clean tillage and two mulching ages (four and eight years), respectively. This plant has strong adaptability, high N fixation capacity, high coverage, a shallow root system, and no need to cut. Specifically, the objectives of this paper include (i) analyzing the impacts of mulching ages and soil layers on enzyme activities and soil nutrients, (ii) revealing the correlation of enzyme activities with soil properties, and (iii) identifying the key factors influencing soil enzyme activities in regards to mulching ages and soil layers.

## Materials and methods

### Study site

Field experiments were performed in a citrus orchard on a sloping site in Zigui County, Hubei Province, China (110°40’ E, 31°4’ N). The local climate belongs to the subtropical monsoon climate. The annual average temperature and precipitation were 16.7 °C and 1013.1 mm, respectively. The soil is purple and mainly has a sandy loam texture. Since the 2000s, citrus has been continuously cultivated in the study area.

The citrus had a planting density of about 825 plants ha^-1^: plant spacing, 3 ± 0.5 m; row spacing, 3.5 ± 0.5 m. The main fertilizer used was mixed fertilizers containing 22% N, 6% P_2_O_5_, and 11%K_2_O (about 3300 kg ha^-1^). The fertilizers were used three times a year, including one base fertilizer and two top dressings.

### Experimental design

A species of *Vicia villosa* (VV) was used as mulching grass in the citrus orchard and sown initially using the full mulching method (45 kg ha^-1^) in September 2013 and 2017, respectively. Then, the grass grew naturally and was not cleaned. The weeds in the citrus orchard with clean tillage were manually removed. Other field management practices (e.g., fertilization type and time) at these experimental sites were the same. Therefore, the experimental site with grass mulching included mulching for four years (VV_4) and eight years (VV_8) by 2021. The experimental site without mulching (i.e., clean tillage) was taken as the control (CT). At each site, every two plots had a distance of more than 50 m. Three replicates were used for analysis (**Table 1**).

**Table 1:** The basic information of experimental plots with living grass mulching.

Plots	Altitude (m)	Slope (°)	Area (m^2^)	Planting density of VV (kg ha^-1^)	Aspect
VV_8	220	20	200	45	south
VV_4	220	20	200	45	south
CT	220	20	200	0	south

### Soil sampling

Soil samples were obtained from citrus orchards with different LGM ages in March 2021. Soils were collected from three soil layers (i.e., 0-10, 10-20, and 20-40 cm) in each plot using a five-point sampling method and combined into one mixed sample by soil layer. A total of 27 soil samples (three mulching ages × three soil layers × three replicates) were collected. Stones and large roots were removed from the fresh soil. Then, the collected soil samples were sieved using a 2-mm sieve and split into halves. One part was stored at 4°C to determine soil moisture content, microbial biomass, and enzyme activities. The other part was naturally air-dried to determine the physicochemical properties of soil samples.

### Soil physicochemical properties and microbial biomass

The soil sample was dissolved into water (soil:water = 1:2.5) to determine soil pH. Total nitrogen (TN), SOC, and total phosphorus (TP) were calculated employing the Kjeldahl method, the K_2_Cr_2_O_7_-H_2_SO_4_ oxidation method, and acid melt-molybdenum, antimony, and scandium colorimetry, respectively (Zheng et al., 2020). Based on the modified alkaline hydrolysis diffusion method and the Olsen method, alkali-hydrolyzed nitrogen (AN) and available phosphorus (AP) were analyzed (Wang et al., 2016). Based on the chloroform fumigation-extraction method, soil microbial biomass nitrogen (MBN), carbon (MBC), and phosphorus (MBP) were measured (Brookes et al., 1985; Vance et al., 1987). Soil moisture content (MC) was measured using the ring sampler method.

### Soil enzyme activities

Six soil enzymes were selected, including *β*-glucosidase (BG), cellobiohydrolase (CB), N-acetylglucosaminidase (NAG), leucine aminopeptidase (LAP), acid phosphatase (APH), and phenol oxidase (POX). Their activities were measured using microplate fluorimetry (German et al., 2011; Zhou et al., 2020). Soil suspensions were obtained by adding fresh soil (equivalent to 1g of dry soil) into 125 mL sodium acetate buffer (50 mmol L^-1^; pH = 5.0-6.6) and stirring for 1 min. Soil suspensions (200 µL) and corresponding enzyme substrates (i.e., 7-amino-4-methylcoumarin [AMC for LAP] and 4-methylumbelliferone [MUB for BG, CB, NAG, and APH], 50 µL, 200 µmol L^-1^) were combined in eight sample assay wells of 96-well microplates. Then, the microplates were incubated in the dark at 25 °C for 3 hours. Fluorescence at 365 nm excitation and 450 nm emission filters was determined based on a microplate fluorometer (SpectraMax i3x, Molecular Devices, Beckman Coulter, CA, USA). The soil suspension (600 µL) and the substrate (L-3,4-dihydroxyphenylalanine, DOPA, 150 uL, 25 mmol L^-1^), were mixed and then added to the 96-well microplates in order to determine the POX activity. The microplates were incubated for an hour with shaking. The absorbance was measured at 465 nm. The activities of the six enzymes were measured in the unit of nmol h^-1^ g^-1^ soil.

The C, N, and phosphorus (P)-cycling enzyme activity and total enzyme activity were calculated using the normalization method. The geometric mean (GM) was calculated to evaluate the enzyme activities with different functions and the total enzyme activities (Eqs. (1-4); Raiesi and Salek-Gilani, 2018):


(1) $$\;\;\text{GMC}\;=\;\sqrt[{\text{n}1}]{\prod_{\text{i}=1}^{\text{n}1}{\text{EC}}_{\text{i}}}$$

(2) $$\text{GMN}\;=\;\sqrt[{\text{n}2}]{\prod_{\text{i}=1}^{\text{n}2}{\text{EN}}_{\text{i}}}$$

(3) $$\text{GMP = }\sqrt[{\text{n}3}]{\prod_{\text{i}=1}^{\text{n}3}{\text{EP}}_{\text{i}}}$$

(4) $$\text{GM}\;=\;\sqrt[{\text{n}1+\text{n}2+\text{n}3}]{\prod_{\text{i}=1}^{n1}{\text{EC}}_{\text{i}}\times \prod_{\text{i}=1}^{\text{n}2}{\text{EN}}_{\text{i}}\times \prod_{\text{i}=1}^{\text{n}3}{\text{EP}}_{\text{i}}}$$

where GMC, GMN, and GMP indicate C, N, and P-cycling enzyme activities, respectively; *EC_i_*, *EN_i_*, and *EP_i_* are the normalized value of enzyme *i* in C, N, and P-cycling, respectively; *n1*, *n2*, and *n3* are the number of enzymes in C, N, and P-cycling, respectively; GM indicates the total enzyme activity. In this study, *n1 =* 3 (POX, BG, and CB), *n2 =* 2 (NAG and LAP), and *n3 =* 1 (APH).

### Statistical analysis

Statistical analysis was performed using R v.3.6.1. First, Tukey’s HSD tests and one-way and two-way analysis of variance (ANOVA) were performed to evaluate the variations of soil enzyme activities, microbial biomass, and physicochemical properties under different mulching ages, different soil layers, and their interactions. Then, Pearson’s correlation analysis was used to determine the relationships of soil enzyme activities and other soil properties. Univariate and multivariate (stepwise) linear regression analyses were performed to determine the magnitude of the interaction between other soil properties and the individual soil enzyme activity. Finally, based on the above information, redundancy analysis (RDA) was used to simultaneously examine all soil enzyme activities and the influence of other soil properties. To remove collinearity among variables, a Monte Carlo permutation test (999 permutations) and variance inflation factor inspection (VIF < 5) were used to identify effective variables. Then, the impact of other soil properties on soil enzyme activity was investigated. The importance ranking of the main influencing factors of soil enzyme activity was further determined using hierarchical partitioning (Lai et al., 2022).

## Results

### Effects of mulching age and soil layer on soil properties

**Table 2** shows the effects of mulching age and soil layer on orchard soil properties. The soil layer significantly influenced all microbial biomass and soil physicochemical properties (*P* < 0.05). The mulching age significantly affected all microbial biomass and all soil physicochemical properties except for TN and TP. Their two-way interaction only significantly affected AP.

**Table 2:** Statistical differences (*F*-values and significance level) between means of soil properties by two-way ANOVA with mulching age and soil layer.

Factor	SOC	TN	AN	TP	AP	pH	MC	MBC	MBN	MBP
Mulching age (A)	6.04^**^	3.46	7.18^**^	1.21	25.24^***^	5.10^*^	40.97^***^	187.87^***^	26.57^***^	14.05^***^
Soil layer (L)	24.11^***^	15.32^***^	13.18^***^	6.84^**^	12.05^***^	4.14^*^	9.71^***^	5.61^*^	82.93^***^	12.69^***^
A*L	0.47	0.55	0.73	1.23	3.82^*^	0.22	0.28	0.16	1.94	2.74

SOC, soil organic carbon; TN, total nitrogen; AN, alkali-hydrolyzed nitrogen; TP, total phosphorus; AP, available phosphorus; MC, moisture content; MBC, MBN, and MBP represent microbial biomass carbon, nitrogen, and phosphorus, respectively. *, **, and *** represent *P* < 0.05, *P* < 0.01, and *P* < 0.001, respectively. The data is *F* value.

**Figure 1:**
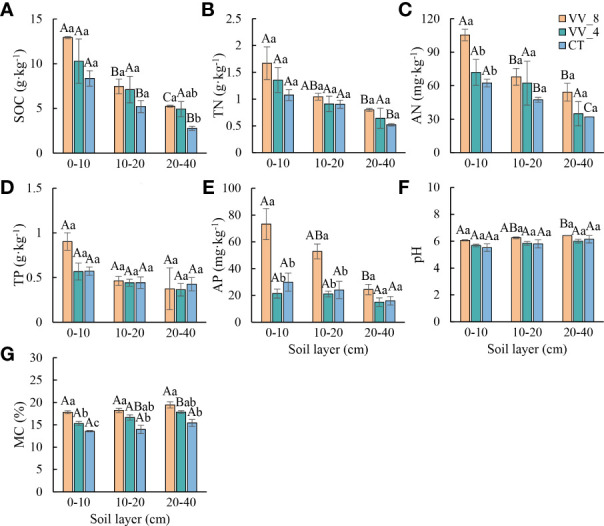
Soil physicochemical properties under different mulching ages within the different soil layers in citrus orchards. **(A–E)** The contents of soil organic carbon, total nitrogen, alkali-hydrolyzed nitrogen, total phosphorus, available phosphorus. **(F)** pH. **(G)** Soil moisture content. Values are the mean ± standard error (*n* = 3). Capital letters represent significant differences in different soil layers under the same mulching age. Lowercase letters represent significant differences under different mulching ages in the same soil layer based on Tukey’s tests and one-way ANOVA (*P* < 0.05). VV_8, mulching for eight years; VV_4, mulching for four years; CT, clean tillage; SOC, soil organic carbon; TN, total nitrogen; AN, alkali-hydrolyzed nitrogen; TP, total phosphorus; AP, available phosphorus; MC, moisture content.

Soil physicochemical properties generally varied with mulching ages in the three soil layers (especially at 0-10 cm) (**Figure 1**). SOC, TN, AN, pH, and MC progressively increased with increasing mulching ages in all soil layers (**Figures 1A–C, F, G**). TP and AP decreased under VV_4 and then significantly increased under VV_8 (**Figures 1D, E**). In comparison to the CT treatment, the VV_8 treatment substantially improved AN (69.08%) and AP (144.96%) at 0-10 cm, SOC at 20-40 cm (89.29%), and MC in all three soil layers (31.29%, 30.58%, and 26.10%) (*P* < 0.05) The VV_4 treatment only significantly increased MC by 12.77% at 0-10 cm (*P*< 0.05). TN, TP, and pH had no significant increase under different mulching ages in each soil layer.

**Figure 2:**
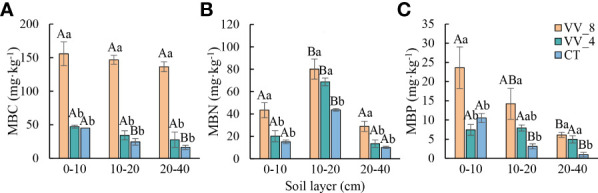
Soil microbial biomass in different soil layers under different mulching ages in citrus orchards. **(A-C)** The contents of soil microbial biomass carbon, nitrogen, and phosphorus. Values are the mean ± standard error (*n* = 3). Capital letters represent significant differences under the same mulching age in different soil layers. Lowercase letters represent significant differences under different mulching ages in the same soil layer based on Tukey’s tests and one-way ANOVA (*P* < 0.05). VV_8, mulching for eight years; VV_4, mulching for four years; CT, clean tillage; MBC, MBN, and MBP represent microbial biomass carbon, nitrogen, and phosphorus, respectively.

MBC, MBN, and MBP also showed an upward trend with mulching ages in all soil layers, except for MBP at 0-10 cm (**Figure 2**). MBC at three soil layers under the VV_4 treatment was slightly higher than that under the CT treatment, while the increase was not significant (**Figure 2A**). MBN at 10-20 cm and MBP at 20-40 cm under the VV_4 treatment significantly increased by 57.36% and 404.50%, respectively, compared to those under the CT treatment (*P* < 0.05; **Figures 2B, C**). MBC, MBN, and MBP under the VV_8 treatment in all soil layers significantly increased by 245.72-733.85%, 83.82-186.57%, and 124.56-522.61%, respectively, compared to those under the CT treatment (*P* < 0.05; **Figure 2**).

The soil microbial biomass and physicochemical properties showed a similar trend with the soil layer (**Figures 1**, **2**). In general, except for pH and MC, all other biochemical properties decreased with the soil layer depth (i.e., 0-10 cm > 10-20 cm > 20-40 cm). Remarkably, MBN was the highest at 10-20 cm (*P* < 0.05).

### Effects of mulching age and soil layer on soil enzyme activities

From **Table 3**, the mulching age significantly affected all soil enzyme activities (*P* < 0.05). The soil layer significantly influenced all soil enzyme activities except for POX. The combined effect of mulching age and soil layer only significantly affected LAP, APH, GMN, GMP, and GM (*P* < 0.05). The mulching age and soil layer significantly affected C, N, and P-cycling and total enzyme activities (*P* < 0.001).

**Table 3:** Statistical differences (*F*-values and significance level) between means of soil enzyme activities by two-way ANOVA with mulching age and soil layer.

Factor	POX	BG	CB	NAG	LAP	APH	GMC	GMN	GMP	GM
Mulching age (A)	9.39^**^	18.18^***^	3.67^*^	57.07^***^	38.67^***^	145.58^***^	29.56^***^	126.91^***^	145.58^***^	132.83^***^
Soil layer (L)	1.02	24.10^***^	28.98^***^	3.84^*^	11.79^***^	28.83^***^	21.43^***^	21.33^***^	28.83^***^	43.42^***^
A*L	0.25	2.50	1.25	0.88	3.32^*^	4.21^*^	2.20	4.80^**^	4.21^*^	7.18^***^

POX, phenol oxidase; BG, *β*-glucosidase; CB, cellobiohydrolase; NAG, N-acetylglucosaminidase; LAP, leucine aminopeptidase; APH, acid phosphatase ; GMC, GMN, and GMP represent C, N, and P-cycling enzyme activities, respectively; GM, total enzyme activities. *, **, and *** represent *P* < 0.05, *P* < 0.01, and *P* < 0.001, respectively. The data is *F* value.

**Figure 3:**
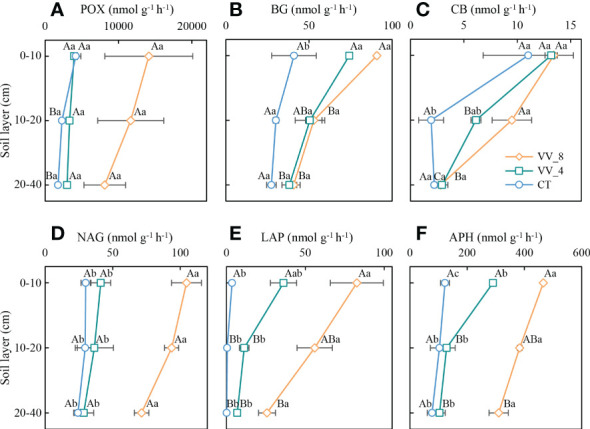
Soil enzyme activities under different soil layers and mulching ages in citrus orchards. **(A–F)** The activities of phenol oxidase, β-glucosidase, cellobiohydrolase, N-acetylglucosaminidase, leucine aminopeptidase, acid phosphatase. Values are the mean ± standard error (*n* = 3). Capital letters represent significant differences under the same mulching age in different soil layers. Lowercase letters represent significant differences under different mulching ages in the same soil layer based on Tukey’s tests and one-way ANOVA (*P* < 0.05). VV_8, mulching for eight years; VV_4, mulching for four years; CT, clean tillage; POX, phenol oxidase; BG, *β*-glucosidase; CB, cellobiohydrolase; NAG, N-acetylglucosaminidase; LAP, leucine aminopeptidase; APH, acid phosphatase.

The soil enzyme activities were ranked in descending order in terms of mulching age and soil layer: VV_8 > VV_4 > CT; 0-10 cm > 10-20 cm > 20-40 cm (**Figure 3**). The BG activity (0-10 cm) and CB activity (10-20 cm) under the VV_8 treatment were 1.22 and 3.90 times greater than those under the CT treatment (*P* < 0.05), respectively. The NAG, LAP, and APH activities under the VV_8 treatment in all soil layers were 1.94-2.49, 22.17-223.77, and 2.71-3.00 times greater than those under the CT treatment, respectively (*P* < 0.05). Furthermore, compared to the CT treatment, the VV_4 treatment only significantly increased the activities of BG (81.16%) and APH (136.77%) at 0-10 cm (*P* < 0.05).

**Figure 4:**
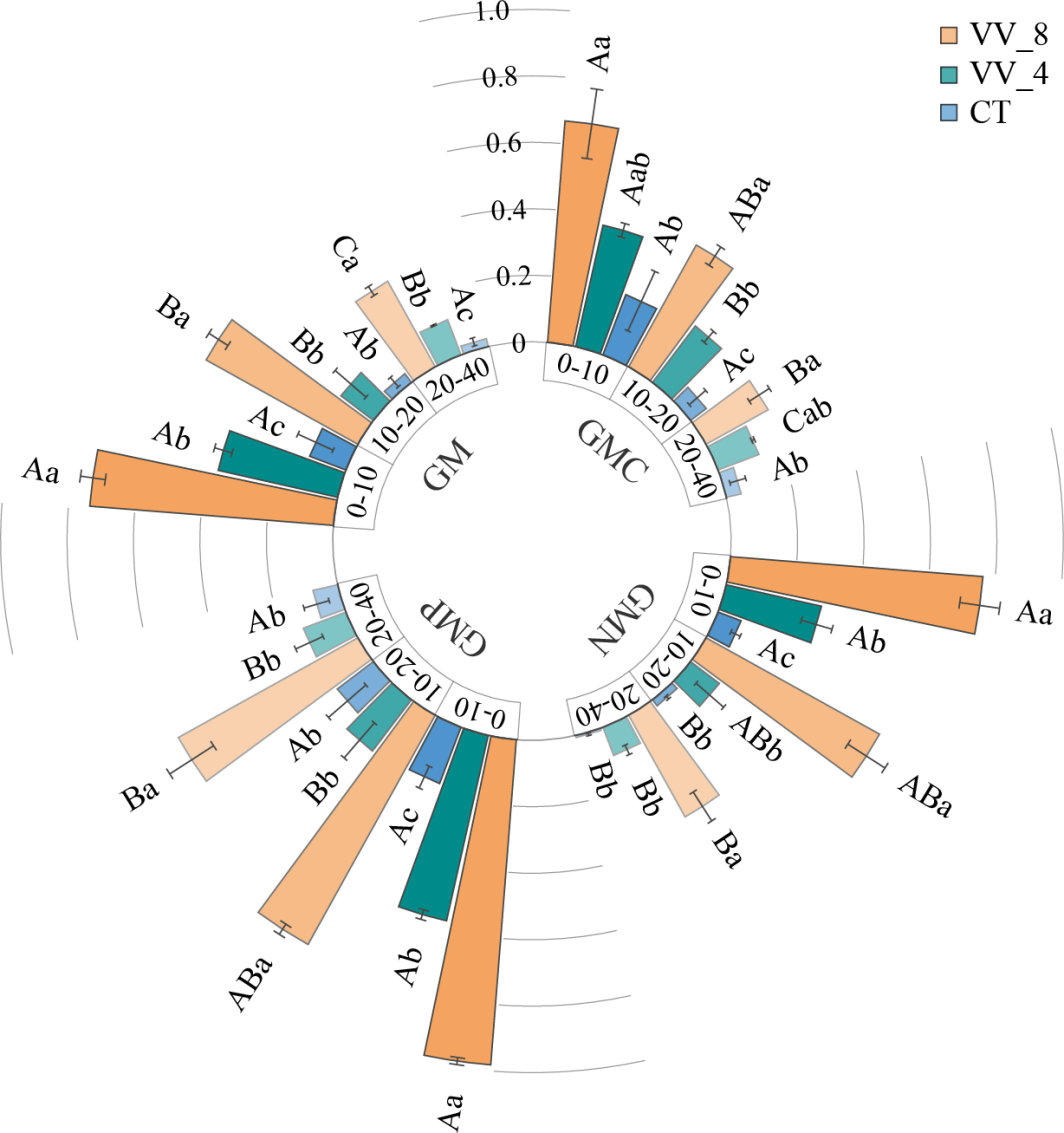
Soil C-cycling (GMC), N-cycling (GMN), P-cycling (GMP), and total (GM) enzyme activity in different soil layers under different mulching ages in citrus orchards. Values are the mean ± standard error (*n* = 3). Capital letters represent significant differences under the same mulching age in different soil layers. Lowercase letters represent significant differences under different mulching ages in the same soil layer based on Tukey’s tests and one-way ANOVA (*P* < 0.05). VV_8, mulching for eight years; VV_4, mulching for four years; CT, clean tillage.

The activities of GMC, GMN, GMP, and GM increased with mulching age in all soil layers. Under the VV_8 treatment, they were 2.45-6.07, 9.29-54.42, 4.42-7.11, and 5.32-14.91 times higher than those under the CT treatment, respectively (*P* < 0.05). The VV_4 treatment significantly enhanced GMC activity at 10-20 cm, GMN and GMP activities at 0-10 cm, and GM activity at 0-10 cm and 20-40 cm by 259.84%, 296.44%, 215.55%, 216.45%, and 360.98%, respectively (*P* < 0.05). GMC, GMN, GMP, and GM activities declined as the soil layer got deeper. Under the VV_8 and VV_4 treatments, these activities were much higher at 0-10 cm compared to those at 20-40 cm. The CT treatment only significantly changed GMN (**Figure 4**).

### Main influencing factors of soil enzyme activity

**Figure 5:**
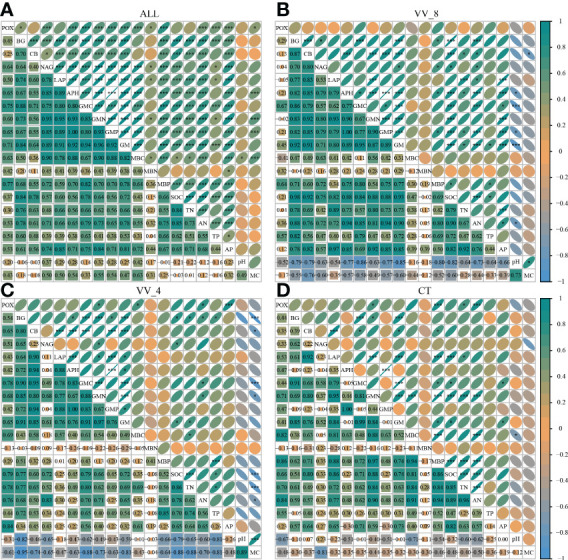
Correlation of soil enzyme activities with other soil properties. **(A)** All treatments. **(B)** The treatment of mulching for eight years. **(C)** The treatment of mulching for four years. **(D)** The treatment of clean tillage. VV_8, mulching for eight years; VV_4, mulching for four years; CT, clean tillage; POX, phenol oxidase; BG, *β*-glucosidase; CB, cellobiohydrolase; NAG, N-acetylglucosaminidase; LAP, leucine aminopeptidase; APH, acid phosphatase; GMC, GMN, and GMP represent C, N, and P-cycling enzyme activities, respectively; GM, total enzyme activities; MBC, MBN, and MBP represent microbial biomass carbon, nitrogen, and phosphorus, respectively; SOC, soil organic carbon; TN, total nitrogen; AN, alkali-hydrolyzed nitrogen; TP, total phosphorus; AP, available phosphorus; MC, moisture content. * and *** represent *P* < 0.05 and *P* < 0.001.

**Figure 6:**
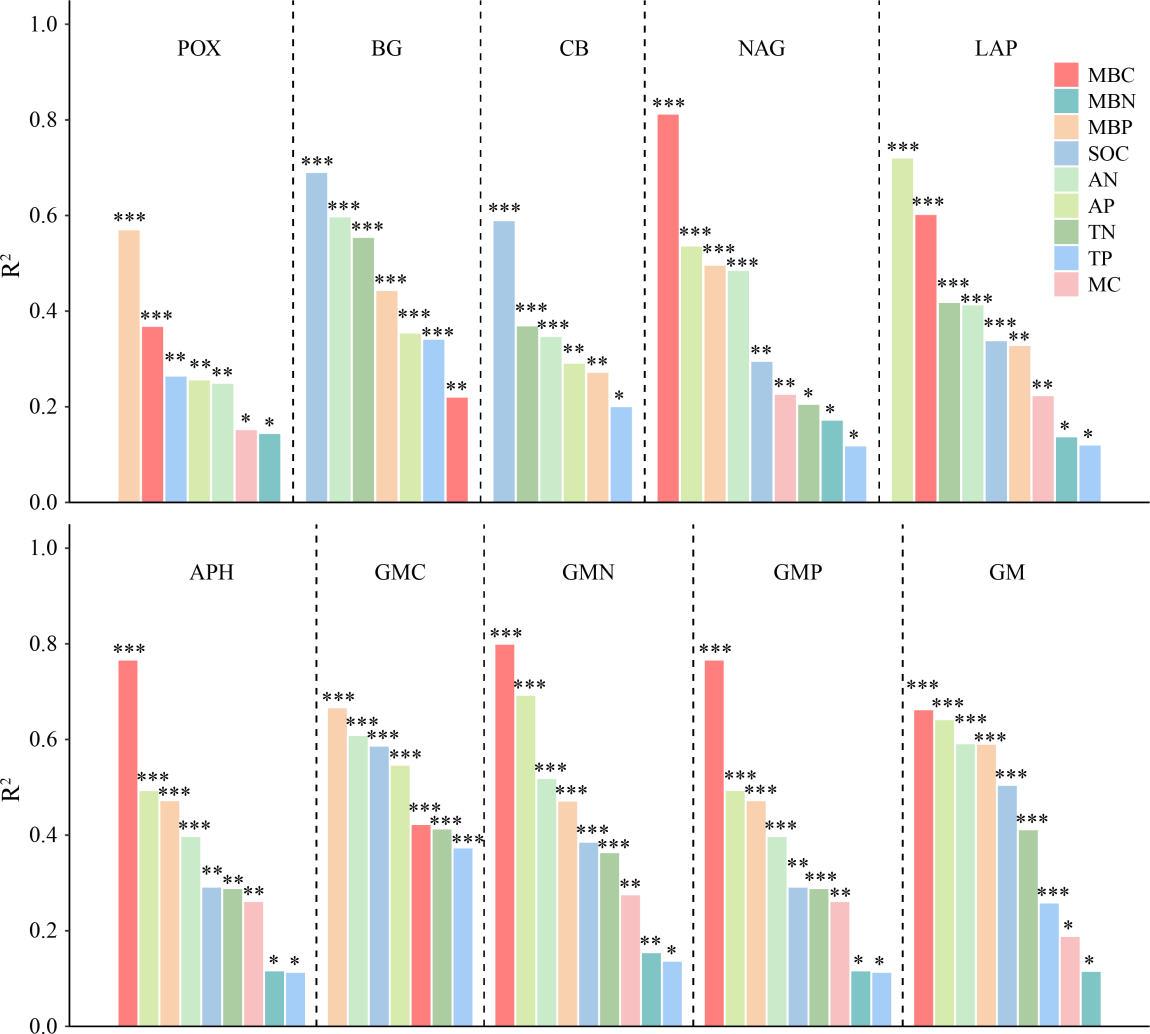
The adjusted R^2^ of univariate regression analysis for soil enzyme activities and soil physicochemical properties and microbial biomass. POX, phenol oxidase; BG, *β*-glucosidase; CB, cellobiohydrolase; NAG, N-acetylglucosaminidase; LAP, leucine aminopeptidase; APH, acid phosphatase; GMC, GMN, and GMP represent C, N, and P-cycling enzyme activities, respectively; GM, total enzyme activities; MBC, MBN, and MBP represent microbial biomass carbon, nitrogen, and phosphorus, respectively; SOC, soil organic carbon; TN, total nitrogen; AN, alkali-hydrolyzed nitrogen; TP, total phosphorus; AP, available phosphorus; MC, moisture content. *, **, and *** represent *P* < 0.05, *P* < 0.01, and *P* < 0.001, respectively.

The correlation analysis reveals that the enzyme activity generally exhibited significant positive correlations with MBC, MBP, AN, and AP (*P* < 0.001). SOC and TN also had a positive and significant correlation with all enzyme activities (*P* < 0.001; **Figure 5A**), except for POX and APH activities. Under the VV_8 treatment, the correlation of BG, NAG, APH, and GMP activities with MBP was significantly positive (*P* < 0.05). The correlation of BG, CB, and GMC activities with SOC was significantly positive (*P* < 0.001). LAP activity showed a significant positive correlation with TN (*P* < 0.001). N and P-cycling enzyme activities exhibited a significant positive correlation with AN and AP (*P* < 0.05; **Figure 5B**). BG activity was positively associated with AN, SOC, TN, and TP under the VV_4 treatment (*P* < 0.05; **Figure 5C**). The enzyme activities exhibited a significant positive correlation with soil C and N nutrients under the CT treatment (*P* < 0.05; **Figure 5D**). Univariate and stepwise regression analyses found similar results to explain the relationship between soil enzyme activities and other soil properties. MBC, MBP, SOC, AN, and AP had significant and strong univariate relationships with each enzyme activity (*P* < 0.01; **Figure 6**). Also, stepwise regression output showed that the standardized regression coefficients of MBC, MBP, SOC, AN, and AP were statistically significant (*P* < 0.05; **Table 4**) and had higher absolute values. These results indicate that the response of soil enzyme activity to MBC, MBP, SOC, AN, and AP was more sensitive, that is, these five biochemical properties had a greater positive effect on soil enzyme activity.

**Table 4:** The standardized regression coefficient of multivariate (stepwise) linear regression analysis.

Variables	R^2^	MBC	MBN	MBP	SOC	TN	AN	TP	AP	pH	MC
POX	0.57***	0.23	0.16	0.77***	0.24	0.17	0.11	0.06	0.03	0.21	0.26
BG	0.69***	0.17	0.08	0.23	0.84***	0.17	0.26	0.14	0.12	0.13	0.20
CB	0.59***	0.04	0.01	0.06	0.78***	0.10	0.18	0.01	0.10	0.14	0.07
APH	0.80***	0.78***	0.05	0.12	0.23*	0.04	0.09	0.003	0.004	0.01	0.10
NAG	0.87***	0.74***	0.07	0.04	0.02	0.15	0.30**	0.01	0.01	0.15	0.01
LAP	0.77***	0.21	0.02	0.02	0.21	0.25	0.19	0.09	0.77***	0.03	0.26*
GMC	0.81***	0.07	0.07	0.50***	0.50***	0.08	0.10	0.06	0.09	0.25*	0.14
GMN	0.90***	0.58***	0.01	0.07	0.09	0.01	0.24*	0.02	0.26*	0.07	0.14
GMP	0.80***	0.78***	0.05	0.12	0.23*	0.04	0.09	0.003	0.004	0.01	0.10
GM	0.83***	0.63***	0.08	0.19	0.46***	0.04	0.20	0.10	0.18	0.13	0.17

POX, phenol oxidase; BG, *β*-glucosidase; CB, cellobiohydrolase; NAG, N-acetylglucosaminidase; LAP, leucine aminopeptidase; APH, acid phosphatase; GMC, GMN, and GMP represent C, N, and P-cycling enzyme activities, respectively; GM, total enzyme activities; MBC, MBN, and MBP represent microbial biomass carbon, nitrogen, and phosphorus, respectively; SOC, soil organic carbon; TN, total nitrogen; AN, alkali-hydrolyzed nitrogen; TP, total phosphorus; AP, available phosphorus; MC, moisture content. *, **, and *** represent *P* < 0.05, *P* < 0.01, and *P* < 0.001, respectively. Numbers in bold font represent the variables entered into the model, and the numbers in regular font represent variables removed from the model.

**Figure 7:**
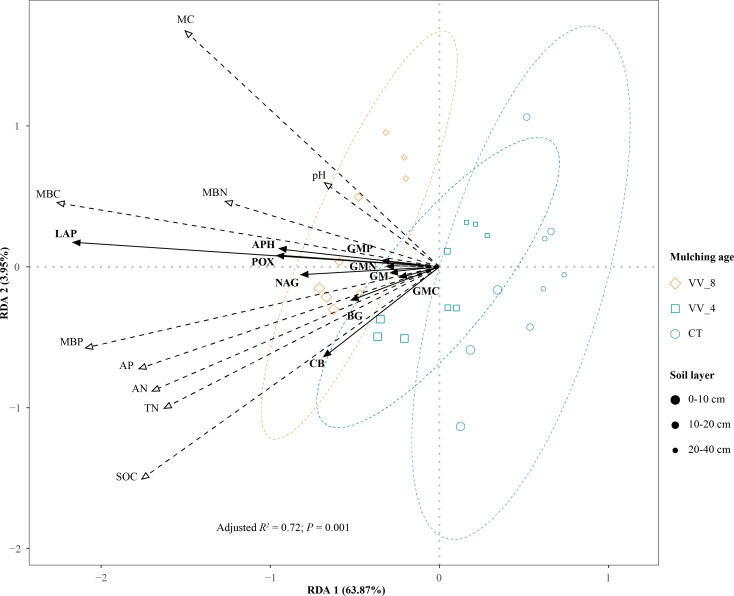
Redundancy analysis (RDA) ordination plot of enzyme activities constrained by physicochemical properties and microbial biomass that significantly explained variation. VV_8, mulching for eight years; VV_4, mulching for four years; CT, clean tillage; POX, phenol oxidase; BG, *β*-glucosidase; CB, cellobiohydrolase; NAG, N-acetylglucosaminidase; LAP, leucine aminopeptidase; APH, acid phosphatase; GMC, GMN, and GMP represent C, N, and P-cycling enzyme activities, respectively; GM, total enzyme activities; MBC, MBN, and MBP represent microbial biomass carbon, nitrogen, and phosphorus, respectively; SOC, soil organic carbon; TN, total nitrogen; AN, alkali-hydrolyzed nitrogen; AP, available phosphorus; MC, moisture content.

The two main axes (RDA) 1 and 2 were selected with an explanation of 63.87% and 3.95%, respectively (**Figure 7**). RDA shows that soil properties showed positive effects on enzyme activities (**Figure 7**). The soil properties were ranked in descending order in terms of importance: MBC (16.40%) > MBP (13.18%) > MC (11.15%) > SOC (9.09%) > AN (6.33%) > AP (5.57%) > TN (5.15%) > pH (3.09%) > MBN (1.54%). The response of soil enzyme activities to the top five soil properties was significant (*P* < 0.05; **Table 5**). MBC had the longest arrow line and the highest explanation amount. This indicates that MBC was the most important influencing factor. The available nutrients that had significant impacts, i.e., MBC, MBP, and AN, accounted for nearly 70% of the indices that significantly influenced soil enzyme activities (**Table 5**).

**Table 5:** Significance test results and importance ranking of soil properties.

Soil properties	Importance	Percentage of soil properties in total variation	*P*
MBC	1	16.40	0.012^*^
MBP	2	13.18	0.012^*^
MC	3	11.15	0.020^*^
SOC	4	9.09	0.016^*^
AN	5	6.33	0.046^*^
AP	6	5.57	0.068
TN	7	5.15	0.064
pH	8	3.09	0.132
MBN	9	1.54	0.212

*indicates *P* < 0.05. MBC, microbial biomass carbon; MBP, microbial biomass phosphorus; MC, moisture content; SOC, soil organic carbon; AN, alkali-hydrolyzed nitrogen; AP, available phosphorus; TN, total nitrogen; MBN, microbial biomass nitrogen.

In general, MBC and MC mainly affected the activities of GMN (LAP), GMP (APH), and POX. MBP mainly affected GM, GMC (BG and CB), and NAG activities. BG and CB activities were also affected by SOC and AN. In addition, the effects of soil properties on soil enzyme activities varied with mulching ages and soil layers. The VV_8 treatment positively affected all enzyme activities at all soil layers, especially at 0-20 cm. The VV_4 treatment only positively affected BG and CB activities at 0-10 cm.

## Discussion

### Effects of different mulching ages on soil enzyme activities

LGM could improve soil nutrients and enzyme activities by increasing SOC input from root exudates and above-ground residues (Qian et al., 2015; Kader et al., 2017). We found that LGM’s effects on enzyme activity were closely correlated with mulching ages and soil layers. The BG and APH activities at 0-10 cm were notably higher under the VV_4 treatment than the CT treatment (**Figure 3**). The BG activity was an important indicator reflecting the quality of organic matter and C sink level (Cenini et al., 2016). BG can hydrolyze cellobiose to generate glucose and provide metabolites for soil microorganisms (Singhania et al., 2013). After short-term mulching with *Vicia villosa*, cellulose and other *β*-1, 4-glucan polymers dominated the soil organic matter input and directly acted on the topsoil. Thus, the secretion of hydrolase enzymes (especially BG) was improved. These enzymes were the most closely related to the organic matter formed by the decomposition of herbaceous residues (Sinsabaugh et al., 2008). This is consistent with the finding of Feng et al. (2021). They reported that a short-term leguminous grass mulching system increased the BG activity compared with the non-mulching treatment, indicating the increased C inputs from grass mulching which could stimulate microbial activity. The increase of APH activity after short-term mulching may be related to P sequestration in herbs and fruit trees. In the initial mulching stage (the VV_4 treatment), soil P decreased at 0-10 cm (**Figures 1**, **2**) due to the competition for P between fruit trees and herbs. This may induce short-term P limitation in the soil (Deng et al., 2017). The result also agreed with the study by Chen et al. (2020). Therefore, fruit trees may release more root exudates to stimulate microbial activity and increase P secretion to alleviate P limitation.

Under the VV_8 treatment, the enzyme activities significantly increased, especially N-cycling (NAG and LAP) and P-cycling (APH) enzyme activities (**Figures 3**, **4**). After long-term mulching with *Vicia villosa*, the enzyme activity was higher due to increased soil organic matter by herbaceous residue accumulation. Increased C input could weaken microbial C limitation and increase unstable components of SOC (Kalinina et al., 2019). Thus, the formation and release of enzymes were accelerated, and enzyme activities were effectively promoted. NAG and LAP were mainly involved in soil N transformation (Cenini et al., 2016) and chitin and peptide decomposition, respectively. The increased activities of these two enzymes may be attributed to the enhanced N fixation capacity of legumes and the weakened P limitation after long-term mulching. The increase in N availability promoted the N-cycling enzyme activities. The increase in the NAG activity may be due to the increase in the number of fungi caused by the long-term accumulation of herbaceous residues (Ramos-Zapata et al., 2012; Wang et al., 2020b) since chitin mainly exists in fungal cell walls and animal exoskeletons (Zheng et al., 2018). Wang et al. (2020b) showed that long-term mulching promoted the increase of NAG activity. However, another study stated that NAG was not directly affected by the decomposition of crop residues. Thus, long-term mulching did not affect its activity (Zheng et al., 2018). In addition, the continuous increase of APH indicates that the soil still showed P deficiency, although P limitation was alleviated after long-term mulching. Therefore, to satisfy tree growth needs, long-term accumulation of herbaceous residues still continuously promoted the conversion of organic P to inorganic P (Singh et al., 2018), thus maintaining a high APH activity.

### Responses of soil enzyme activities in different soil layers to living grass mulching

The enzyme activity generally decreases with the deepening of soil layers (Stone et al., 2014). The topsoil is more conducive to promoting enzyme activity than the subsoil (Uksa et al., 2015; Avazpoor et al., 2019). The enzyme activities under LGM in this study also decreased with increasing soil depth (**Figures 3**, **4**), which agreed with the findings of Sun et al. (2021). The result was related to grassroot distribution and nutrient input of surface residues. The grassroots were mainly distributed between 0-20 cm (Liu et al., 2018). The effect of surface residues on soil nutrients (i.e., C turnover and N and P mineralization) directly acted on the topsoil (Moradi et al., 2017). Therefore, the activities of five hydrolase enzymes (except for NAG) under LGM were significantly lower in the subsoil than in the topsoil (**Figures 3**, **4**). POX activity had no significant changes among different soil layers.

Under LGM, the difference in soil enzyme activity at different soil layers may be due to various interactions between enzymes and microbial populations. LGM could increase the overall C metabolic activity due to increased soil organic matter input (Qian et al., 2015). This enhancement effect may be related to increased soil bacteria. The increase of microbes may be associated with certain enzyme secretion (i.e., BG and CB), thus enhancing soil C-cycling. Moreover, the N-fixation of the legume herb mulching promoted an increase in soil N metabolism and stimulated protein production of N-cycling bacterial communities. Thus, the activities of related enzymes, such as LAP, were enhanced. Some studies have shown that the alpha diversity of bacteria was lower in the subsoil than in the topsoil due to decreased oxygen and a low-nutrient environment in the deep soil (Wang et al., 2020a). Therefore, the enzyme activities were related to increased bacteria and were much higher in the topsoil than in the subsoil. As mentioned above, NAG mainly hydrolyzed chitin secreted by fungal cell walls. However, related studies have shown that fungal alpha diversity was not significantly different among soil layers (Wang et al., 2020a). Fungi may have high adaptability to LGM-induced environmental changes. This was one of the possible reasons for the insignificant differences in the NAG activity at different soil layers. In addition, the correlation between the enzyme activity and enzyme producers was weaker for oxidase than hydrolase (A’ Bear et al., 2014). Thus, the POX activity had no significant differences at soil layers.

### Factors mediating soil enzyme activity under living grass mulching

Soil enzyme activity is influenced by biotic and abiotic factors (Jian et al., 2016), such as soil nutrients, microbial biomass, and moisture content. Under LGM, soil organic matter increased with the continuous input of root litter and surface residues (Wei et al., 2017). The increased soil organic matter could promote microbial activity and extracellular enzyme secretion. Thus, soil enzyme activity had a positive correlation with organic matter. In this study, SOC had the most significant positive correlation with the C-cycling enzyme activity. This indicates that SOC was the key factor influencing the C-cycling enzyme activity (**Figures 5**, **7**). Compared to TN, AN also significantly positively affected BG and CB activities. The results show that available nutrients had stronger effects than total nutrients. Sun et al. (2021) also found that soil enzyme activity was more easily affected by available nutrients. Conversely, some studies have found that the C-cycling enzyme activity was significantly positively affected by TN (A’Bear et al., 2014; Qian et al., 2015; Zheng et al., 2018).

In this study, N and P-cycling enzyme activities were mainly positively affected by MBC and MBP, followed by AN (**Figure 7**). The result indicates that some biotic factors (e.g., microbial biomass) were more critical to soil enzyme activities. The soil microbial biomass is not only a key and highly active pool for storing soil nutrients but also a sensitive microbial activity indicator to reflect soil quality (Muñoz et al., 2017). Under long-term mulching, surface residues can provide microorganisms with sufficient metabolic substrates, promote the absorption and utilization of C, P, and other elements by microorganisms, and then accelerate the secretion of N and P-cycling enzymes. Some studies also found that MBC was positively correlated with soil enzyme activity (Bowles et al., 2014; Sun et al., 2021).

We also found that MC positively affected soil enzyme activities (**Figure 7**). Moisture is an essential determinant of soil enzyme activity, which increases with soil MC (Baldrian et al., 2013). LGM could enhance soil porosity and promote water infiltration and storage (Blanco-Canqui et al., 2011; Basche et al., 2016). Soil structure gradually improved with mulching ages. The increase in enzyme activity may be correlated to the enhancement of permeability and agglomeration ability (Roldán et al., 2005). In addition, soil moisture is essential in maintaining MBC (Kader et al., 2017). This study demonstrated a significant positive correlation between MBC and MC (**Figures 5**, **7**). Therefore, the positive effect of MC on enzyme activity may be attributed to the mediating role of MBC.

In addition, except for soil physicochemical properties, soil enzyme activities were also affected by other factors, e.g., climate, soil type, and management measure. Previous studies have found that soil enzyme activities can be affected by climate, i.e., temperature and precipitation (Zhou et al., 2013; Jian et al., 2021). The effects of temperature on soil enzyme activities were directly correlated with the variations in the kinetic characteristics of enzymes (Steinweg et al., 2013). The increase in temperature can generally enhance C and N-cycling enzyme activities (Wallenstein et al., 2009). However, some studies have shown opposite conclusions (A’Bear et al., 2014). Soil moisture closely related to precipitation was positive with enzyme activities, which was another important factor affecting enzyme activities (Steinweg et al., 2012). Soil enzyme activity was also affected by soil type, which may be closely related to unique soil properties, such as texture (Acosta-Martínez et al., 2007; Štursová and Baldrian, 2010; Zhang et al., 2017). Moreover, management measures, such as fertilization and mulching materials and methods, can also affect soil enzyme activity in the orchard ecosystem (Kader et al., 2017; Zheng et al., 2020). Previous studies found that green organic manure significantly increased soil enzyme activity compared to inorganic fertilization (Piotrowska and Wilczewski, 2012). Orchard grass (Gramineae) with high C/N can improve the C-cycling enzyme activity while grass (Leguminosae) with low C/N can promote the N-cycling enzyme activity (Wang et al., 2020b). The mixture of legumes and other grasses was more conducive to promoting soil enzyme activity compared to single grass mulching (Chavarría et al., 2016). In this study, consistent management measures were adopted at all of the experimental plots to avoid the impact of human interference and then highlight the effect of mulching age on soil enzyme activities. This study focused on the influence of soil biological and physicochemical properties on enzyme activities. In our previous research, the effects of cultivated grass (*Vicia Villosa*) and natural grass (*Galium spurium* and *Stellaria media*) on soil enzyme activities were preliminarily compared. The results show that the improvement of soil enzyme activities by leguminous grass mulching was higher than that by natural grass mulching (Wang et al., 2023). This result emphasized the importance of selecting appropriate grass types in the orchard ecosystem. Therefore, in future research, different influencing factors should be further studied to implement more sustainable practices.

## Conclusion

In general, long-term mulching (the VV_8 treatment) effectively improved soil nutrient and enzyme activity levels. However, short-term mulching (the VV_4 treatment) had fewer effects on soil biochemical properties. The improvement effect of living grass mulching on soil enzyme activities was soil layer-dependent. Long-term mulching can affect deeper soil layers than short-term mulching. Compared to other soil properties, available nutrients (i.e., MBC, MBP, and AN) had significant effects on enzyme activities. Thus, soil enzyme activities could be improved through enhanced available nutrients. In addition, this study indicates that increasing nutrient availability by extending mulching age can be an effective strategy for sustainable soil management in orchard systems.

## Data availability statement

The original contributions presented in the study are included in the article. Further inquiries can be directed to the corresponding author.

## Author contributions

NW: conceptualization, investigation, data curation, methodology, formal analysis, visualization, writing - original Draft, writing - review and editing. LL: formal analysis, visualization, writing - review and editing. MG: data collection, writing - review and editing. ZJ: investigation, writing - review and editing. JH: investigation. HC: visualization. WX: resources, supervision, project administration. CL: conceptualization, supervision, project administration, funding acquisition. All authors have read and approved the manuscript.
